# The Future of Precision Medicine in the Cure of Alzheimer’s Disease

**DOI:** 10.3390/biomedicines11020335

**Published:** 2023-01-25

**Authors:** Azher Arafah, Saima Khatoon, Iyman Rasool, Andleeb Khan, Mashoque Ahmad Rather, Khaled Abdullah Abujabal, Yazid Abdullilah Hassan Faqih, Hina Rashid, Shahzada Mudasir Rashid, Sheikh Bilal Ahmad, Athanasios Alexiou, Muneeb U. Rehman

**Affiliations:** 1 Department of Clinical Pharmacy, College of Pharmacy, King Saud University, Riyadh 11451, Saudi Arabia; 2 Department of Medical Elementology and Toxicology, School of Chemical and Life Sciences, Jamia Hamdard, New Delhi 110062, India; 3 Department of Pathology, Government Medical College (GMC-Srinagar), Karan Nagar, Srinagar 190010, India; 4 Department of Pharmacology and Toxicology, College of Pharmacy, Jazan University, Jazan 45142, Saudi Arabia; 5 Department of Molecular Pharmacology & Physiology, Bryd Alzheimer’s Research Institute, Morsani College of Medicine, University of South Florida, Tampa, FL 33620, USA; 6 Department of Pharmacy, Abu Areish General Hospital, Abu Areish 45911, Saudi Arabia; 7 Department of Emergency and Accident, Abu Areish General Hospital, Abu Areish 45911, Saudi Arabia; 8 Division of Veterinary Biochemistry, Faculty of Veterinary Sciences and Animal Husbandry, Sher-e-Kashmir University of Agricultural Sciences and Technology (SKUAST-K), Srinagar 190006, India; 9 Novel Global Community Educational Foundation, Hebersham, NSW 2770, Australia; 10 AFNP Med, Haidingergasse 29, 1030 Vienna, Austria

**Keywords:** precision medicine, Alzheimer’s disease, biomarkers, neuroimaging, artificial intelligence

## Abstract

This decade has seen the beginning of ground-breaking conceptual shifts in the research of Alzheimer’s disease (AD), which acknowledges risk elements and the evolving wide spectrum of complicated underlying pathophysiology among the range of diverse neurodegenerative diseases. Significant improvements in diagnosis, treatments, and mitigation of AD are likely to result from the development and application of a comprehensive approach to precision medicine (PM), as is the case with several other diseases. This strategy will probably be based on the achievements made in more sophisticated research areas, including cancer. PM will require the direct integration of neurology, neuroscience, and psychiatry into a paradigm of the healthcare field that turns away from the isolated method. PM is biomarker-guided treatment at a systems level that incorporates findings of the thorough pathophysiology of neurodegenerative disorders as well as methodological developments. Comprehensive examination and categorization of interrelated and convergent disease processes, an explanation of the genomic and epigenetic drivers, a description of the spatial and temporal paths of natural history, biological markers, and risk markers, as well as aspects about the regulation, and the ethical, governmental, and sociocultural repercussions of findings at a subclinical level all require clarification and realistic execution. Advances toward a comprehensive systems-based approach to PM may finally usher in a new era of scientific and technical achievement that will help to end the complications of AD.

## 1. Introduction

In ageing populations, the prevalent type of dementia is Alzheimer’s disease (AD) and it affects nearly 30 million elderly people worldwide. By 2050, it is anticipated that this number will have quadrupled due to the high ageing population that will create a significant load on healthcare systems [1,2]. Currently, AD is effectively incurable because the medications that are currently available have a negligible impact on the severity and progression of the disease. The impact on public health would be greatly reduced, and individuals suffering from the effects would be reduced by interventions that prevent, stop, or slow the progression of the disease.

The failure of current treatments for AD can be attributed to a few factors. One is that current treatments are primarily symptomatic, meaning they only address the symptoms of the disease and do not address the underlying causes. Additionally, these treatments are not effective for all patients and can have side effects. The side effects of current treatments for AD can include gastrointestinal issues, such as nausea and diarrhea, as well as sleep disturbances and changes in behaviour. These medications can also have interactions with other medications that a patient may be taking. Another factor that contributes to the failure of current treatments is that they are typically prescribed at a late stage of the disease when significant brain damage has already occurred. This makes it difficult for the treatments to be effective.

**Figure 1:**
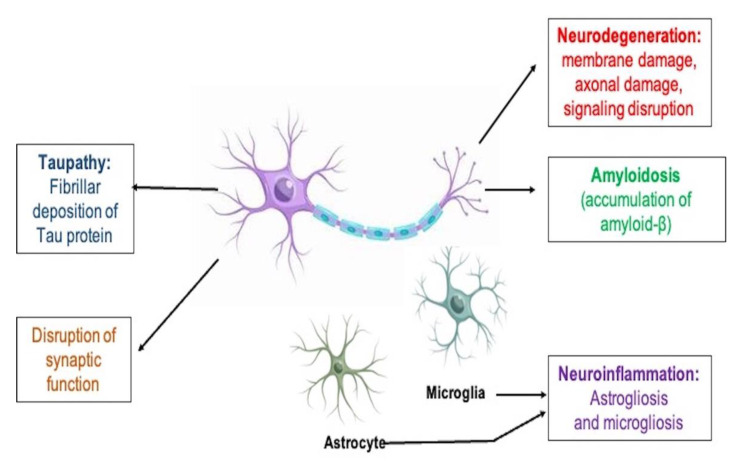
Biological indicators of AD’s histopathological changes.

As a result, there is a compelling need to create pharmacological treatments that can stop the disease from progressing in its early phases when there is still significant neural and cognitive capability [3]. Drug families that are currently on the market, such as acetylcholinesterase inhibitors and non-competitive N-methyl-D-aspartate (NMDA) antagonists, provide symptomatic relief and are only administered following the clinical identification of dementia [4]. AD has a range of pathological hallmarks, including extracellular amyloid beta (Aβ) buildup, neuronal cell degeneration, and intracellular aggregation of tau protein that led to the development of neurofibrillary tangles (NFTs) [5]. Growing evidence from clinical and pre-clinical research on the common involvement of inflammatory processes in AD, as well as other neurodegenerative illnesses, has revealed an extensive range of therapeutic options for the disease’s prevention and therapy [4] because important mediators of disease aggravation such as chemokines, cytokines, astrocytes, and microglia comprise the innate immune response known as the neuroinflammation [6]. Neuroinflammation and microglial activation play an important part in the Aβ and tau aggregation concept of the AD [7]. Pathological events that occur in the pathogenesis of AD are illustrated in Figure 1.

According to estimates, postponing the initiation of AD by five years may cut its occurrence by almost half [2]. Complex chronic diseases with widespread unmet requirements including tumors, diabetes, immunological disorders, and brain proteinopathies including AD, are principally marked by the confluence of polygenetic, epigenomic, genomic, interactomic, and environmental vulnerability and predominantly show: (I) a multifactorial disposition; and (II) disrupted networks influencing relevant circuits and interactomes [8,9]. The failure of ongoing late-stage clinical drug trials that were primarily created under the outdated model for drug development in AD serves as evidence that a compositional move in drug discovery and development programs is essential to achieve effective, innovative improvements in novel therapeutics [10]. The overall influence of a compound on four important points including (I) striking the desired target, (II) affecting the anticipated mechanisms, (III) modifying the underlying mechanisms, and (IV) influencing the clinical result is particularly crucial for the development of effective medications [8].

Through the application of PM, numerous healthcare sciences including oncology and cardiology, have been improved [10]. The goal is to significantly spread awareness of the importance of this strategy in order to transform the current healthcare system completely. The manuscript discusses the mechanisms involved in AD and therapeutic approaches influencing the disease, then moves on to the crucial elements of PM, which may be an important component of AD drug development. This encourages neurodegenerative investigators to concentrate on a clear, individualized strategy to help manage AD.

## 2. Concept of Precision Medicine

Precision Medicine Initiative was established by the National Institute of Health (NIH) in 2015 and numerous other organizations involved in research as a novel method of tackling medicine with a focused and patient-specific approach [11]. These institutes have stated specifically that “developing approaches for treating and preventing disease that take into account individual heterogeneity in the environment, genes, and lifestyle for each person” constitutes PM [11]. This method of practicing medicine has a great deal of potential for addressing the unique characteristics of people with various lifestyle, genetic, and associated comorbidities that could alter their reaction to therapy. Numerous specialities, including cancer and cardiology, have started to devote their efforts toward a more precise means of executing medical procedures since its introduction [12,13].

Following earlier attempts to classify and combine disease states and treatment possibilities based on the individual diagnosis, PM is now being used in the clinical system [14]. Examples include using blood type to expedite blood transfusions or choosing appropriate antibiotics based on the drug sensitivity of pathogenic bacteria when diagnosing and treating phenylketonuria in newborns. Some examples include checking for specific gene alterations in the BRCA2 and BRCA1 genes in breast cancer patients or customising cystic fibrosis treatment to target a specific cause related with the patient’s illness [14]. Numerous high-throughput strategies of characterizing patient biomarkers have been combined with vital advancements in computer-based approaches necessary for assessing the substantial quantity of data created by such techniques to enable the implementation of PM in numerous therapies [14]. The core component of major government programmes to change medical practice is the notion of PM, which explains the widespread impact of increased information on the complex developments connected with a person’s health and for forecasting the effectiveness of therapy [14].

**Figure 2:**
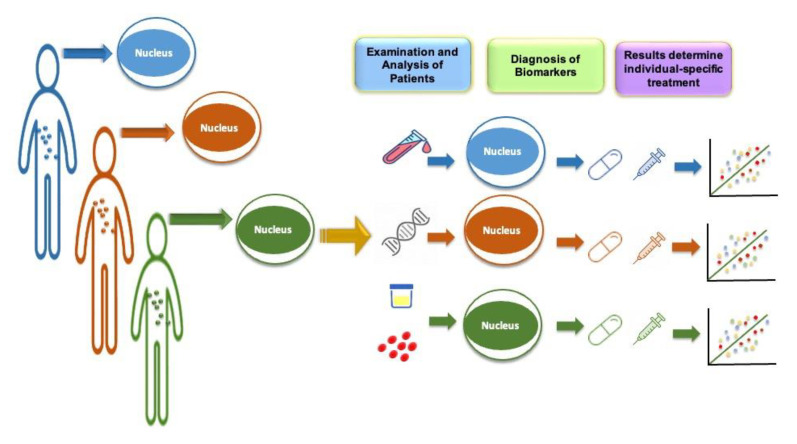
Precision medicine approach to prevention of disease. Precision medicine in Alzheimer’s disease involves the use of biomarkers, such as genetic and imaging markers, to accurately diagnose and classify patients based on the specific subtype of the disease they have. This allows for more tailored treatment approaches and the development of targeted therapies.

Late onset Alzheimer’s disease (LOAD) is a form of AD that develops after the age of 60. It is the most common form of the disease and accounts for the majority of cases. Risk factors for LOAD include age, genetics, and lifestyle factors such as smoking, high blood pressure, and a lack of physical activity. Numerous studies have examined the part that genetics has to play at the start of LOAD; one study estimated that genetics accounted for over 50 percent of the phenotypic variable [15]. The transition from generic risk-lowering strategies to specific interventions focusing on particular risk variables, notably genetics, has not yet been fully accomplished in the field of AD prevention. In this scenario, the complementary roles of genomic studies, investigation and analysis of fluid-based biological markers, and multi-modal brain imaging will enable the identification of distinct biological mechanisms and signaling cascades in symptomless individuals at the highest threat for development to clinical benchmarks. Due to the field speculation that initial biomarker-driven personalized therapies may present the best possibility of therapeutic achievement, genomic research has led to the identification of genetic risk factors for Alzheimer’s disease, which can aid in early detection. This paradigm shift is moving away from the traditional “one size fits all” concept in drug research. This will make it possible to recognize and describe disease states at the undetected preclinical phase, where pathophysiology and topographic anomalies occur many years to decades before extreme clinical signs. The transition in brain research and AD toward biomarker-directed, “molecularly” tailor-made treatment for highly effective prevention and treatment options are made possible by the PM strategy. The concept of PM is demonstrated in Figure 2.

## 3. Alzheimer’s Disease and Other Related Disorders

### 3.1. Alzheimer’s Disease

Alzheimer’s disease is a progressive neurodegenerative disorder that affects memory, thinking, and behaviour. It is the most common cause of dementia in older adults. The exact causes of Alzheimer’s are not fully understood, but a combination of genetic, lifestyle, and environmental factors likely play a role. Symptoms of the disease typically develop slowly and worsen over time, eventually impairing a person’s ability to carry out daily activities. There is no cure for Alzheimer’s, but medications and other therapies can help manage symptoms and improve quality of life. Research is ongoing to better understand the disease and to develop new treatments. Neuroinflammation, or inflammation in the brain, is thought to play a role in the development and progression of Alzheimer’s disease. Studies have shown that there is an increase in inflammatory markers in the brains of individuals with Alzheimer’s, and that this inflammation is associated with the accumulation of amyloid plaques and tau tangles, which are hallmarks of the disease. Some researchers believe that the neuroinflammation seen in Alzheimer’s may be caused by an abnormal immune response to the amyloid plaques and tau tangles, while others suggest that chronic, low-grade inflammation in the brain may contribute to the development of the disease.

Microglial responses may have a negative impact on AD because they produce reactive nitrogen species (RNS) and reactive oxygen species (ROS). As a result, the neuronal cells may die due to oxidative stress, which may be followed by chronic stress [16]. TNF-α has been shown to be linked to initial proinflammatory mechanisms in AD by human longitudinal studies and animal studies [17,18]. Early pro-inflammatory processes and the lack of anti-inflammatory cytokines, such as TGF β1, have been linked in preclinical AD models to a significant neurobiological association in AD patients [19]. TGF β1 (neurotrophic factor) deficiency is a significant factor in the development of AD [4]. Additionally, it demonstrates a crucial role in the formation of memory and synaptic plasticity, supporting the transition from early to subsequent long-term potentiation (LTP) [20]. PM approaches to target LTP in AD might include drugs that enhance LTP by modulating specific signaling pathways in the brain, such as the one mediated by N-methyl-D-aspartate (NMDA) receptors, which play a crucial role in LTP. Additionally, drugs that target the formation of amyloid plaques or abnormal accumulation of tau protein, which are hallmarks of AD and may interfere with the normal functioning of synapses and disrupt LTP, may also be beneficial. Furthermore, genetic profiling of patients with Alzheimer’s disease may help identify those who are most likely to respond to LTP-targeted therapies, and thus precision medicine approaches to target LTP may be more effective when tailored to the specific needs of individual patients.

The neuroinflammatory mechanisms could be disrupted with neuroprotection, which is brought on by brain-derived neurotrophic factors (BDNF), as well as impaired neurotrophin signaling [21]. Neuroinflammation, which thus plays a crucial role in AD, interferes with the maturation in addition to the functioning of nerve growth factor (NGF) [4]. According to research in transgenic and animal AD models, the pro-inflammatory processes that start before the deposition of plaque and are facilitated by solubilized Aβ oligomers impede the NGF metabolic pathway, which is linked to a delayed transition of precursor pro-NGF to mature NGF [22]. Metalloprotease-9 (MMP-9) is overactive in the neural tissues of AD patients due to neuroinflammation [22]. Increased MMP-9 activity would thus promote the breakdown of mature NGF, which would subsequently compromise the ability of mature NGF to maintain the trophic dependency of cholinergic neuronal cells [23].

PM approaches that target pro-inflammatory or anti-inflammatory molecules in AD may have the potential to slow the progression of the disease or improve outcomes for patients. For example, drugs that target the action of specific pro-inflammatory molecules, such as TNF-α, may be able to reduce the inflammatory response and slow the progression of the disease. Additionally, therapies that increase the levels of anti-inflammatory molecules may also be beneficial. However, it is important to note that more research is needed to fully understand the role of these molecules in AD and the potential for PM approaches targeting them.

**Figure 3:**
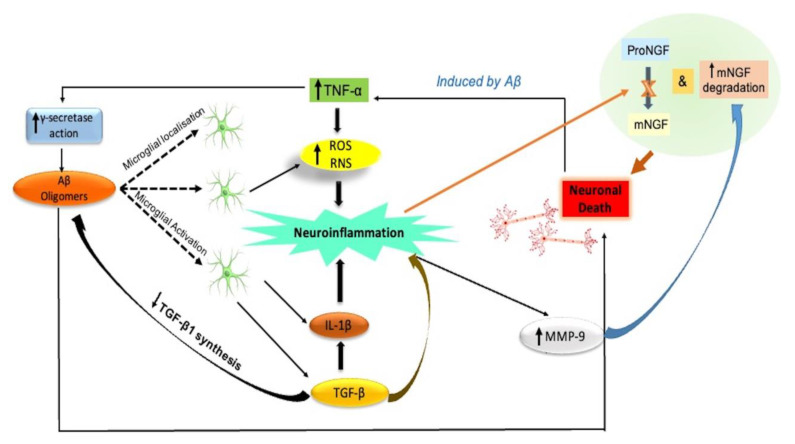
Neuroinflammation: A key initiating component in the development of AD. Signaling of neurotrophins is impaired. By inducing microglia to release proinflammatory cytokines (IL-1β and TNF-α) and blocking the production of anti-inflammatory cytokines such as TGF-β1, Aβ oligomers cause neuroinflammation and neuronal death in the AD brain. TNF-α promotes g-secretase activity and prevents microglia from phagocytizing Aβ, which promotes Aβ build up and microglia-mediated neuroinflammation. Through the production of ROS and RNS, proinflammatory microglial activities also accelerate the death of neurons. Eventually, neuroinflammatory phenomena may play a role in the pathogenesis of AD by impairing neurotrophin signaling. This includes decreasing the synthesis of TGF-β1 and impairing the NGF metabolic pathway, which is characterized by a decreased conversion of proNGF to biologically active mNGF and a higher rate of mNGF degradation aided by MMP-9. Aβ, amyloid beta; IL-1b, interleukin-1 beta; MMP-9, metalloprotease-9; NGF, nerve growth factor; mNGF, mature nerve growth factor; proNGF, precursor of the nerve growth factor; RNS, reactive nitrogen species; ROS, reactive oxygen species; TGF-β, transforming growth factor-beta; TNF-α, tumor necrosis factor-alpha.

The neuroinflammatory process is illustrated in Figure 3.

### 3.2. Parkinson’s Disease

With a prevalence of 0.3% in developed nations, the second most common neurodegenerative condition after AD is Parkinson’s disease (PD). It is standard to diagnose PD clinically using impaired motor function. Bradykinesia, rigidity, tremor, and postural instability are the key symptoms, with an asymmetric initiation that eventually spreads to become bilateral [24]. Neurodegeneration in the substantia nigra pars compacta and the eventual diminishing of striatal dopamine content are recognized as being mainly accountable for the standard PD features. The pathogenesis of the Lewy body is unidentified, but the finding that misfolded α-synuclein is a significant component of radiating filaments and is also evident in neuronal function as Lewy neurites has modified views on their creation and involvement in neuron damage and causes a significant change in considering about the emergence and advancement of the disease from a pathophysiological viewpoint, leading to the grouping of PD as a synucleinopathy [25]. Nearly 17 autosomal dominant and autosomal recessive gene mutations are known to cause different forms of familial PD, according to research [26]. Parkin, ubiquitin carboxyl-terminal hydrolase, phosphatase and tensin homolog-inducible kinase 1, leucine-rich repeat kinase 2, and glucocerebrosidase are a few of these. The theories causing the loss of dopaminergic neurons in PD continue to be founded on the idea of oxidative stress. Oxidative stress may be brought on in PD by changed iron buildup, changed proteolysis, altered calcium channel function, altered α-synuclein aggregation, and the existence of mutant proteins [27,28,29]. There is proof of widespread systemic inflammation in PD, signifying that it may, in several cases, be the major cause of neuronal loss. Microglia stimulation and inflammatory changes were previously assumed to be a result of neuronal damage. Furthermore, peripheral inflammation may intensify the negative consequences of an inflammatory process in the substantia nigra [30].

### 3.3. Dementia with Lewy Bodies

Dementia with Lewy bodies and PD are the clinical conditions that constitute Lewy Body Dementia (LBD). It is a slowly progressing form of Parkinsonism with psychosis, dementia, and other hallmarks. Symptoms change with time and differ from individual to individual. It is distinguished by the accumulation of Lewy bodies, intraneuronal cytoplasmic inclusion bodies with clumps of ubiquitin and α-synuclein in the brain [31]. PD and AD share pathologies with LBD. Lewy bodies (ubiquitin and α-synuclein aggregates) are neuronal cytoplasmic inclusion bodies that are diagnostic of Lewy body dementia and are present in the brain parenchyma, particularly in the limbic system, brainstem, and cerebral cortex [32]. Environmental toxins, genetic mutations, and the aging process can cause misfolding of α-synuclein and its accumulation in the form of Lewy bodies via oxidative stress and mitochondrial dysfunction.

### 3.4. Amyotrophic Lateral Sclerosis

Adult-onset neurodegenerative condition ALS (amyotrophic lateral sclerosis) is a serious condition. In this regard, a number of hypotheses have been put out, and it appears likely that a number of pathways, rather than just one, are involved in the neurodegeneration seen in ALS, indicating multifactorial pathophysiology. Both sporadic and familial ALS patients’ skeletal muscle and spinal motor neurons, as well as the murine ALS model, showed morphological changes in mitochondria, including vacuolated and swollen organelles with disorderly cristae and membranes, fractured network, and edema [33,34,35]. The increase of the mitochondrial intermembrane gap and subsequent membrane distention causes vacuoles to form [36]. Additionally, ROS in ALS may result from improper oxidative phosphorylation [37], according to research on the CSF of transgenic mice and humans, where significant concentrations of ROS caused by improper oxidative phosphorylation, including 3-nitrotyrosine, were discovered [38]. Patients with ALS had increased oxidative stress in their CSF, urine, and serum [39], that may be caused by a modified geometry in the mutant SOD1’s active region that permits lowering substrate entrance. The developments of ALS and neurodegeneration have both been linked to reactive astrogliosis. Injured motor neurons cause microglial cells to emit ROS and inflammatory cytokines, develop an M1 phenotype, and increase neurotoxin production as the disease progresses [40]. Studies on astrocytes in the post-mortem tissues of ALS patients found that 22 genes producing proinflammatory cytokines, chemokines, and elements of the complement cascade were upregulated. This could accelerate neuronal death and the loss of already impaired neurons [40].

## 4. Precision Medicine Application to AD

It is not a new idea to use a PM paradigm to develop creative treatments, preventative measures, and therapeutic cures for complex diseases. Despite the fact that the oncology community spent years grappling with how to treat patients who ultimately passed away from late-stage advanced tumours, today’s mortality and treatment rates—especially for certain types of cancer—are significantly higher than prior expectations. On the other hand, despite more than a century of scientific progress, there is still no therapeutic treatment for AD, which is still 100% deadly. Only late, perhaps irreversible clinical illness stages are approved for the currently available treatments, which only provide modest clinical advantages. The discipline of cancer presents a radical shift methodology that has been successfully applied to adopt a PM at this moment [41].

The idea behind PM is to customise medical care to each patient’s unique genetic, physiological, and clinical aspects of the disease [42]. It tries to personalise illness prevention and therapy to the unique biological make-up of the individual (customised treatment), which contrasts sharply with the current “one pill fits all” approach. Given the extreme complexity of AD, it is unlikely, at best, to find a single medication that will meaningfully treat every patient. Other fields, such as oncology and cardiology, are affected similarly. For the PM to be used effectively, it is imperative to incorporate the investigative, interdisciplinary, and cross-disciplinary systems perspective of SB (systems biology), backed by system neurobiology [43,44]. With a focus on drug target identification, validation, and assay development, SB permits a system-level aspect of drug discovery that takes into consideration the whole complication of disease pathophysiology. Oncology and cardio–vascular medicines are two advanced translational research domains of biomedicine that have had great success in recent years with biomarker-guided therapy techniques. Traditional reductionistic categorical nosologies for “neurodegenerative illnesses” represent late-stage clinical phenotypes and syndromes that are fragmented and have various or overlapping histological patterns. Despite ongoing working group attempts to improve categorical criteria for diagnosis, especially after incorporating biomarkers as part of the criteria, the diagnostic accuracy and reliability have been improved [45], and there are some limitations to the present categorical diagnoses systems for neurodegenerative illnesses. A move in the right path has been made with the recent introduction of impartial, agnostic biochemical categorization for dementia and neurological disorders to diagnose and estimate risk in healthy, older people. Long before the onset of the first clinical signs, it is intended to detect the whole spectrum of the specific biochemical abnormalities in aged adults at risk [41]. It is anticipated that the use of PM in the fields of neuroscience, psychiatry, and neurology would result in a paradigm shift in the approach to the treatment of brain illnesses toward early detection and effective early interventions. Before any significant disease progression has taken place, prevention techniques can be used, with a strong emphasis on personalised care. One of PM’s key objectives is to introduce new paradigms for the early detection, classification, diagnosis, therapeutic interventions, and preventative measures of neurodegenerative illnesses based on unique physiological characteristics, as mirrored by multidimensional potential biomarkers [43,46,47]. In this context, investigations in neurogenetics and neuroepigenetics have provided evolving findings of AD biomarkers over the last 20 years [48,49], neurochemistry [50]—having been done both on cerebrospinal fluid (CSF) [51,52] and blood [53,54]—additionally in structural, functional, and metabolic imaging [55,56], and neurophysiology [57].

Innovative biomarker studies are projected to identify specific diagnostic, prognostic, and predictive biomarker characteristics, mimicking the oncology approach, in conjunction with SB, in order to individually tailor the therapy to patients [41]. Additionally, the current “trial-and-error” approach to pharmacological interventions is eliminated by biomarker-guided PM, which has important medical ramifications for patients and healthcare organisations [58]. The definitive objective of PM is to enhance both the standard of patient care and clinical consequences, according to the Institute of Medicine (IOM) Committee Recommendations for Advancing Appropriate Use of Biomarker Tests (Companion Diagnostics) for Molecularly Targeted Therapies [59].

### 4.1. Precision Medicine for AD and the Role of Genetics

A PM strategy to AD treatment will require the complete use of genomics to offer individualised advice. This section covers the influences of genes on late-onset AD as well as demonstrations of a targeted PM approach that focuses on such genetic influences.

#### 4.1.1. APOE Gene

One of the most recognized genetic factors is APOE for late-onset AD [60] and encoding for apolipoprotein E (APOE) protein [61]. At the APOE locus, there are three main polymorphisms: ɛ2, ɛ3, and ɛ4. According to studies, the APOE genotype has a big influence on the AD risk. In particular, the ɛ4 allele has been connected to an increased risk of AD [62], while there is less risk linked with the ɛ2 allele [63]. Additionally, compared to people with just one single copy of the ɛ4 allele, those with two copies had an even higher risk of getting AD [64]. Why APOE ɛ4 is associated with a higher risk of AD, while APOE ɛ2 is associated with a lower risk of AD may be explained by a number of pathophysiologic mechanisms. Firstly, the alleles encode proteins with various molecular characteristics, which affect how APOE binds with Aβ. This differential binding may be responsible for the increased build-up of plaques of Aβ which is the key hallmarks of AD, that was seen in APOE ɛ4 individuals [65]. They differ in how well they attach to and transfer lipids due to their various molecular characteristics. Studies have shown that the progression of atherosclerosis, one of the key risk reasons for AD, is considerably influenced by the associations of the APOE allele including both LDL and HDL receptors [61]. It may be crucial to include this genetic makeup in the AD preventive approach because the ɛ4 allele has been recorded to be 27.3 percent of delayed AD risk and because new research suggests that possible risk-reduction therapies may be selectively efficient (or even less efficient) based on the existence of the ɛ4 allele [66]. Depending on the APOE genotype, a variety of AD preventive strategies can be tailored. Within investigations of APOE ɛ4 allele, it was revealed that a substantial variance in some treatments compared with score of control specifically for people with “ɛ4 alleles”, even though the FINGER trial showed insignificant changes in cognition features among APOE genotypes with multimodal routine modifications [67]. This implies that there may be some innate difference between people who carry the APOE ɛ4 allele and those who do not, which may have affected how well the interventions worked. Trials with a big sample size and greater statistical power are required to ascertain the influence of APOE on multimodal treatments. The APOE genotype can be utilised to target AD preventative therapies, according to other single-factor research studies. A systematic review of the investigations that changed the dietary fat revealed that, in 15 of the trials, people with the APOE ɛ4 allele saw the greatest fluctuations in LDL, HDL, and total cholesterol [68]. In a different study, research reported that, in the Mediterranean diet response, both individuals with or without the APOE ɛ4 allele showed that improved cognitive performance is reflected by the Mini Mental State Exam (MMSE), and only individuals without the 4 allele mainly contributed to the clock drawing test, an evaluation of executive functioning and spatial reasoning [69]. Another analysis revealed that in ɛ4 homozygotes, aerobic fitness was associated with better cognitive function [70]. Likewise, with omega-3 fatty acids, on non-impaired people, three RCTs with ɛ4 alleles demonstrated an increase in cognitive performance with DHA administration [71]. Generally, genotype-specific approaches may help individuals by means of methodology and adopting specifically targeted strategies that have been shown to be most effective for people with a similar genotype. More investigation will be required to reveal the influence of APOE on various physical activities, dietary choices, and lifestyle changes as the PM approach to AD prevention evolves.

#### 4.1.2. MTHFR Gene

Another possible genetic factor for AD is the MTHFR gene, which is easily orderable in commercial labs by doctors, that encodes methylenetetrahydrofolate reductase protein. In the literature, a number of MTHFR polymorphisms were described [72]; however, C677T and A1298C have undergone the most scrutiny as to their relationship to AD [73]. Additionally, it appears that the occurrence of these polymorphisms in the overall population is in height [74]. A study found at least one of these MTHFR polymorphisms was present in 92.5 percent of its AD individuals [73]. The catalytic contribution that MTHFR protein has a rate-limiting role to play in homocysteine to methionine conversion, and that vitamin B (cobalamin and folate) act as cofactors, may be related to the link connecting AD and MTHFR polymorphisms [75]. Homocysteine contributes to inflammation, and it has been linked to memory loss and a higher risk of AD [75,76]. A five-year rate of cognitive decline and baseline homocysteine levels were found to be inversely associated in a study of cognitively healthy persons [75]. A research report of 1000 persons from the Framingham cohort that concentrated on baseline non-impaired individuals discovered a similar relationship among dementia and baseline homocysteine possibility up to 11 years later [76]. A long-term study found that raising homocysteine levels ranging between 10 mg/L and 20 mg/L was related with an 88% higher incidence of cognitive impairment across ten years [77]. Higher serum homocysteine levels occur from alterations in the MTHFR molecule that affect its enzymatic activity, such as the A1298C and C677T polymorphisms [78], thereby having the potential to raise the overall risk of AD. In contrast to the C677T polymorphism, some studies have linked the A1298C polymorphism to an increasing AD risk [79]. Nevertheless, a different investigation revealed that the haplotype C genotype, which combines the two polymorphisms to an additional A1793G polymorphism, is linked to a lower risk of AD [80]. Thus, more investigation concerned with connecting these polymorphisms and the AD risk is necessary. MTHFR genetic status may enable tailored AD preventive therapies, much as APOE. Folic acid, cyanocobalamin, and vitamin B6 supplementation has been found to decrease cognitive deterioration in people with high homocysteine [81,82]. It has been investigated in several trials if reduced homocysteine can affect brain disease and/or cognitive function by using a mixture of B vitamins [81]. Despite the paucity of available data, people who have one or more MTHFR polymorphisms might profit from advice tailored to their genotype. For example, since people with specific MTHFR polymorphisms have diminished catalytic capacity of MTHFR protein, shifting from conventional B vitamins to their own methylated counterparts which do not necessitate hepatic transformation to active metabolite, such as methylcobalamin for cyanocobalamin and methyltetrahydrofolate [5-MTH] for folic acid, may improve outcomes. Another study revealed that 5-MTH administration significantly raised serum folate content as compared to folic acid in people with A1298C and C677T polymorphisms, but it had no effect on serum homocysteine concentration [83]. Therefore, more research examining the relationship between precise MTHFR polymorphisms and the risk of AD and the effects of methylated B vitamins may enhance the domain of PM for AD prevention.

#### 4.1.3. Presenilin 1 and 2 Gene

It would be obvious that mutations only partially explain early-onset AD in light of the finding of different pathogenic variants in amyloid precursor protein (APP) [84]. Four separate studies provide another AD linkage area at 14q24 just one year after the initial APP mutation was discovered [85]. The relevant gene (PSEN1) and the first mutation that caused AD were discovered three years later by researchers [86]. PSEN1 encodes a highly conserved polytopic membrane protein and is essential for intramembrane interaction [87]. PSEN1 mutations cause the accelerated generation of Aβ-42 in the APP. The increasing frequency of Aβ42/Aβ40 indicates that the mutations change where the γ-secretase cleaves APP [88]. There are 10 protein-coding exons in the PSEN1 gene. Additionally, it contains two to three extra exons that code for the 5′-untranslated sites. There has been evidence of alternative splicing of exon-8 in this gene [86].

Based on the available information, PSEN2 is found shortly after PSEN1. PSEN1 and PSEN2 are comparable from a genomic and protein perspective [89]. Late-onset AD is a result of PSEN2 mutations. The condition will advance more slowly than it would in the case of an APP or PSEN1 mutation. The PSEN-2 gene has 10 exons that code for proteins and two additional exons that code for the 5′-untranslated region. PSEN-2 and PSEN-1 are structurally similar, although PSEN-2 has mutations at different codons than PSEN-1. According to reports, only roughly one-third of instances of AD that are dominantly inherited are associated with known mutations in the APP or PSEN genes. It suggests the presence of additional disease loci [90].

Variants in PSEN1 and PSEN2 are also linked to early-onset AD [91]. The catalytic components of γ-secretase, presenilins, are intramembrane proteases. Mutations in presenilins encourage the development of cleavage products like Aβ [92]. Although these episodes are still only self-reported, and although no persistent observation for electrographic (focal) seizures has even been undertaken entirely in this patient population, AD patients with the most common PSEN2 mutation (N141I) had a significant prevalence of seizures (32 percent) [93]. In addition, PSEN2 is particularly relevant to investigate AD hyperexcitability since some PSEN2 mutations are linked to decreased penetrance [94], such that episodes can unintentionally be misclassified as sporadic AD [95]. To define the additive effect of ageing and seizures on disease load in AD, PSEN2 seems interesting. Firstly, PSENs might have a more significant impact on the neuropsychiatric symptoms [96]. Secondly, a major factor driving neuroinflammation is PSEN2 [97]. Canonical γ-secretase function is disrupted by the loss of normal PSEN2 function, which encourages a pro-inflammatory phenotype driven by microglia and inflammatory cytokines [98].

#### 4.1.4. Genome-Wide Significant (GWS) Susceptibility Loci

Numerous loci linked to complex traits have been effectively mapped by genome-wide association studies (GWAS). These connections may provide insight into the molecular mechanisms that are altered in typical complex diseases and pave the way for the identification of novel targeted therapies. The Alzheimer’s Disease Genetics Consortium brought together whites with European ancestry, Japanese, African Americans, and Israeli Arabs for the transethnic GWAS for late-onset AD in the Stage 1 sample that was undertaken by Jun et al. Using condensed results from the GWAS dataset for the International Genomics Alzheimer’s Project, Stage 1 suggestive results from new loci were followed up on. Single-nucleotide polymorphism (SNP)-based testing found genome-wide significant (GWS) relationships for the SNPs in USP6NL/ECHDC3, PFDN1/HBEGF, and BZRAP1-AS1, as well as for the interaction of the APOE 4 allele to NFIC SNP. Additionally, they acquired GWS proof of a novel locus called TPBG’s gene-based connection in the entire sample. The results demonstrate the usefulness of transethnic research for finding new AD susceptibility loci [99].

A different study found that different brain and blood cell types express genes relevant to AD. In blood from 5257 Framingham Heart Study individuals and in brain supplied by 475 Religious Orders Study/Memory & Aging Project participants, genome-wide cis-expression quantitative trait locus (eQTL) mapping was carried out. A word for the interaction between the expressions of “proxy” genes that distinguish one cell type from another is included in cell-type-specific eQTL (ct-eQTL) models. In addition, 11,649 and 2533 more significant gene-SNP eQTL pairs in the blood and brain, respectively, were not found in general eQTL analysis but were discovered by ct-eQTL analysis. It is noteworthy that apoptosis and Wnt signaling pathways were enriched in 386 distinct target eGenes with significant eQTLs shared between blood and brain. These linked genes include five recognised AD loci. The finding that a considerable fraction of GWS ct-eQTLs map within 1 Mb of documented AD loci and that 58 percent of the most important eGenes in these eQTLs have earlier been involved in AD support the potential significance and applicability of successful outcomes in myeloid cell types for AD. This research provided more insights into the role of myeloid cells in AD risks, revealed cell-type-specific expression profiles for known and potentially new AD genes, and uncovered potential novel blood and brain AD biomarkers that highlight the need for cell-type-specific investigation [100].

### 4.2. Role of Physiological Biomarkers in Precision Medicine for AD

A broad spectrum of metabolic pathway indicators must be created, validated, and incorporated into clinical medicine for PM to be fully realised. In this regard, it is necessary to elaborate on the prospective and significant role of blood-based biomarkers in PM for AD.

The key to PM is biomarker-based categorization of therapeutic intervention, with medications such as trastuzumab and imatinib serving as the prototype after exhibiting remarkable success for particular patients [101]. Few other fields of medicine have kept up with the number of diagnostic tools developed for cancer therapy. However, new research suggests that blood-based biomarkers can be used to fully realise the potential of PM paradigms for a variety of disease states which include but are not confined to hypertension [102], multiple sclerosis [103], allergic diseases [101], idiopathic pulmonary fibrosis [104], and diabetes [105].

In AD, a great deal of research has been done on blood-based biomarkers for diagnostic purposes [106,107], prediction of risk [108], and recognising the pathobiology’s complexity [109,110]. The most critical requirement in the field of PM is for companion diagnostic assays (CDA) that helps in identifying which patients are often likely to react to particular therapies but also to help exclude people who might have safety and tolerability concerns [58]. The vast improvements in “omics” technology and analytic capability present innovative potential for the creation of CDA for a variety of disease states.

Along with the well-known Aβ and tau pathophysiologies, AD involves a wide variety of other pathophysiological processes, such as inflammation, immune function, mitochondrial dysfunction, neurotrophic impairment, and impaired redox status. In addition, it appears that in sporadic late-onset AD, Aβ and mechanisms related to tau do not happen in isolation or without interacting with other intracellular or extracellular pathways. As it was before in cancer biomarker-based guided therapy, the intricate network of pathophysiologies via time, location, and system aspects of disease in the brain challenges the notion that “one medication fits all” [58]. Screening biological processes linked to AD may therefore reveal unique treatment approaches [111,112]; in particular, inflammatory pathways might be such targets [113,114]. Various studies have connected inflammation to the pathogenesis of AD [115,116]. Variations to the immune system and inflammation have been linked to the pathophysiology and risk of AD [6,117,118]. Non-steroidal anti-inflammatory medicine (NSAID) long-term use is accompanied with lower risk of acquiring AD [119]. Numerous clinical trials using NSAIDs for the prevention or treatment of AD have been performed, based on clinical, pathobiological, and epidemiological data [120,121]; however, all fell short of meeting the objectives of clinical trials. Numerous genetic variations have been identified by large-scale GWAS that may be related to neuroinflammation. The majority of these genes are involved in synaptic activity, cytokines, intracellular signaling and lipid metabolism. Proteomic studies show that TGF-β, TNF-α, IL-1β, and TREM2 (a receptor protein) are all implicated in the activation and maintenance of a wide variety of interrelated aberrant molecular pathways that are responsible for neuroinflammation. Brain inflammatory endophenotypes and biomarkers tracking various biochemical pathways can help to unravel the temporal aspects between inflammatory responses and other pathophysiological aspects of AD. Large-scale clinical trials evaluating anti-A protofibrils, anti-TREM2 antibody, anti-CD33 antibody, and novel NSAIDs, are expected to benefit from robust biomarker-drug codevelopment pipelines that are involved, actively or passively, on inflammatory targets and showing potential disease-modifying effects. The next step is to develop tailored therapy strategies to target neuroinflammation within the context of PM by leveraging cutting-edge, multimodal tools in conjunction with a systems biology strategy [4]. Furthermore, molecular markers may be used to identify particular disease state subgroups (i.e., endophenotypes) [102] that might be more likely to profit from particular treatments. This method also revealed another subset of instances that were most likely not benefiting and had negative reactions (i.e., the deterioration of cognition). As demonstrated by cancer research, biomarkers can be used to categorise exact patient subsets that are likely to get benefitted from treatment strategies and to differentiate them from patient populations that are most likely to not profit from the similar treatment in AD and neurodegenerative diseases [102]. To be able to (i) provide proof-of-concept and (ii) supply sufficient data for CDA-driven clinical trials, these more recent blood-based forms can also be used to bio repository samples from unsuccessful trials. This is because there is an ever-growing graveyard of flawed AD therapeutic agents which never proceeded past Phase III trials.

Moreover, it is commonly accepted that in the near future, Phase III AD studies for innovative disease-modifying candidate medications will likely be successful. However, without a PM-guided strategy, the research will face a similar conundrum to that of prescribing disease-modifying anti-rheumatic medications, where these medications are largely recommended by experimentation [122]. Regarding costs and clinical outcomes, this strategy is ineffective. If blood-based reports of inherent biological abnormalities can be developed to distinguish those patients that are more expected to react or even adversely (for example, inflammation occurrences) to such disease-modifying treatments, this would have a significantly greater impact on patient outcomes and medical costs than to use pharmacogenomics alone. In addition, rheumatoid arthritis-related CDA tests are now being developed. The solubilized or aggregated forms of “amyloid” or “tau” are also currently removed from the brain by these disease-modifying treatments; however, it is still feasible that more tau or amyloid forms are much more important to the pathogenesis and progress in particular subpopulations of people. On the other hand, one may readily see a near future in which therapies are CDA-guided to specific kinds of amyloid for certain categories of people based on the advancements in cancer research. The CDA should also offer a framework of Phase 2 and 3 trials. The design of clinical trials and CDA for compounds will change as the PM approach for AD develops.

The generation of PM concepts and CDA for AD using profiles and algorithms poses a variety of problems and additional difficulties that have not yet been effectively addressed. The very first pre-analytical recommendations for blood-based markers in AD have recently been created by the international blood-based biomarker working group [123]. To completely comprehend the precision and accuracy of the analysis on therapeutic response, variations, and other characteristics of the device’s functionality, CDA, however, introduces a necessity [124]. In current years, an abundant deal of work is put forth into transitioning CSF-based AD biomarkers from laboratory use only to in vitro diagnostics (IVDs) and laboratory developed tests (LDTs). However, the majority of research in the field of blood-based biomarkers is still being perforned on discovery-based platforms, which are unlikely to ever be converted into LDTs, much less IVDs. Additionally, while bridging between a clinical trial assay and the CDA to be used for introducing medicines with FDA approval, there are statistical difficulties to take into account [125]. Despite these difficulties, the creation of a PM paradigm for AD is still possible thanks to blood-based biomarkers, which present an appealing and significant possibility.

PM’s use of biomarkers offers a strategic potential for technology advancements to enhance human health and lower healthcare costs. The idea of PM is to tailor therapies to specific patients or patient subgroups based on using disease-specific biomarkers. Regarding the general effectiveness of this customised method for locating treatable molecular targets, there is still much discussion. Genomics, molecular imaging, metabolomics, proteomics, and next-generation sequencing are only a few of the many analytical methods available today.

All monomeric variants of Aβ, particularly Aβ42, that form aggregations of conformational species which are bioactive and probably start AD toxicity, must be produced by BACE1 (beta-site amyloid precursor protein cleaving enzyme (1). BACE1 levels and activity rates are elevated in the brains and bodily fluids of AD patients, providing evidence for the idea that BACE1 is crucial to the pathogenesis of AD. BACE1 has additional substrates external to the amyloidogenic way that might be vital for synaptic growth and its homeostasis. There may yet be a safe and effective drug with high substrate specificity, a more precise dosing regimen, patient population, and illness stage. The function of Aβ and BACE1 in physiological functions and important pathophysiological pathways of AD should be the focus of future study. Further research is needed on the roles of BACE1 and its homolog BACE2, as well as the physiology of Aβ in glia and neurons. The biological actions of these crucial enzymes will be clarified by cellular and molecular analyses of BACE1 and BACE2 knockout mice in conjunction with biomarker-based human studies, which will assist in pinpointing their targets and downstream consequences. Such investigations will have significant effects on BACE1 inhibition as an AD treatment strategy [126].

T-Tau, p-Tau, and Aβ-42 are the key biomarkers that have demonstrated greater consistency and should be employed in clinical practise and research. These biomarkers have 85–90% accuracy (Table 1).

### 4.3. Evolving Conception of Neuroimaging in AD Precision Medicine

Using the outcomes of clinical trials as a basis for treatments that patients receive have now been categorised by the measurement of bindings by PET substances particular to the proposed treatment, such as anti-tau and anti-amyloid, and would be able to target treatment to the phase of the disease. Imaging pathological observations such as neurofibrillary tangles (NFTs) and fibrillary A may be used to image pathological insights in vivo. Quantitative analysis has made it possible to validate cognitive evaluations with the precision required for secondary preventive methods in the subclinical phase of the disease. Imaging of tau may make it possible to build novel risk-stratification metrics or possibly use “liquid biopsy” as the foundation for enrolling patients in even earlier therapies that target the tau infrastructure. Aβ imaging has pushed conventional AD clinical trial measurements to a different frontier. It is still unknown, as was said above, if customised tau or amyloid therapies will be required for particular types of proteins amongst particular sub-populations. Should that occur, the use of PET imaging can determine their presence or absence, and CSF testing will be required to direct intervention programs to the patient in question.

The final diagnosis of AD has traditionally been recognised as the identification, positioning, and measurement of characteristic neuropathological alterations in the post-mortem brain. To help the diagnostic procedures in clinical practise, volumetric magnetic resonance imaging (MRI) techniques and fluorodeoxyglucose-PET (FDG-PET) are now only useful after the appearance of symptoms, which typically represent significant disease progression [128].

Novel diagnostic approaches that make it possible to non-invasively image pathogenic and metabolic results in AD may aid the development of medicines that target either Aβ or tau as well as the assessment of disease processes [129]. NFTs linked with tau and extracellular Aβ plaques are notable neuropathological findings. The anterior and posterior cingulate, frontal, lateral, and parietal temporal cortices are the areas where these plaques are most commonly detected; this is a typical distribution that can be utilised to visually interpret PET scans. According to the progression of later clinical symptoms, the visual and the sensorimotor cortex are protected from Aβ plaques until relatively later in the development of AD [130]. Pattern-based studies of the existence and course of cognitive decline and the potential for PM techniques have not yet been shown useful. Compared to patients with prodromal AD of the hippocampus type/mild cognitive impairment related to AD or fully developed AD dementia, the cortical absorption of Aβ is poor in healthy control participants. However, cortical Aβ binding is elevated in a significant portion of elderly people who are cognitively healthy. This conclusion is corroborated by postmortem histopathology data, which reveals that preclinical AD is likely present in up to 30% of non-demented elderly people over the age of 75 who have Aβ plaques. Population-based investigations have not been performed yet, thus they cannot provide information on such base rates for an individual or population. The idea that removing this amyloid will lower the chance of developing AD in elderly people with normal cognitive function is being tested in ongoing studies. Given the significant pathological comorbidity linked to the existence of tau and amyloid, it is highly probable that a PM strategy of combined drugs that target other dysfunctional systems (such as neuroinflammation, mitochondrial dysfunction, neurotrophic dysfunction, and increased lipid peroxidation) in addition to disease-modifying treatments will have a significantly greater clinical impact. In contrast to cerebral hypometabolism (FDG-PET/CT), which is the next most sensitive measure, Aβ-plaque deposition can be identified by amyloid-specific imaging agents for PET/CT as early as fifteen years even prior to the beginning of AD symptoms. These utmost sensitive cognitive measurements, such as episodic memory, are predicted to diminish 10 years before PET/CT [131].

Despite the fact that documented research have shown that a negative Aβ examination accurately predicts the lack of AD, and can therefore be used for identifying patients for anti-amyloid treatment trials [132], it will not be possible to detect for AD by means of PET scans for treatment decision-making in clinical practise unless people have been classified for risk using the other method having good accuracy [133], while having poor prognostic accuracy or specificity [134]. CSF assessments are currently repeatable and shown in standard Kaplan-Meier curves [135] and in disease progression models [136], to categorise poor prognosis and they appear to identify Aβ1-42 peptide decreases about 25 years prior to the start of symptoms. As a result, CSF can be used to categorise persons starting at 40 years of age to be directed for testing with PET/CT given the prevalence of AD around age 75 and the high cost. Blood-based biomarker innovation would undoubtedly be more desired and widely applicable for baseline screenings of bigger populations for much more focused secondary examinations using imaging and CSF technology.

Research uses the cortico/cerebellar standardised uptake value ratio (SUVr) as an indicator of Aβ deposition in AD patients, controls, and comparison groups. As alternate or supplementary reference areas, the pons and centrum semiovale are two more cortical regions that are now being considered. The SUVr determines the ratio between the cerebellum, used as a reference, and a set of cortical regions-of-interest (ROI). Using regional and global cortico-cerebellar SUVr, quantitative analyses may provide an unbiased, quantifiable value to improve the visual read to distinguish cognitively healthy people from AD patients [137,138].

In the initial stages of AD, quantitation also allows for enhanced sensitivity. Of the participants in Aβ-PET investigations conducted by Rodrigue and colleagues, 137 people were in good cognitive health. Global and segmental cortico-cerebellar SUVr were derived, with each hemisphere having eight cortical areas and a cerebellar hemisphere reference that did not include the cerebral peduncles. With advancing years, cortical Aβ deposition gradually increased. For instance, 1.22 as the cut-off SUVr for the best clinically relevant separation from minor deposition of Aβ, 20% of patients older than 60 years demonstrated increased Aβ deposition. Growing older, APOE 4 carrier status and inverse relationships between cognitive processing performance, learning and memory, and verbal inference were all shown to have direct associations. There was no connection between episodic memory and Aβ intake. These results lead the authors to draw the conclusion that patients with increased cortical Aβ may experience cognitive difficulties even early in the development of AD [139].

SUVr was the essential parameter; nevertheless, the visual assessment of grey-white matter separation may become challenging due to variations in resolution, picture noise levels, and reconstruction methods between various PET or PET/CT equipment [140]. When baseline and two-year follow-up SUVr values were evaluated, subjects who were Aβ positive at baseline presented a substantial improvement in SUVr, indicating advanced Aβ deposits. However, at the other side, participants who tested negative for Aβ at baseline did not have a rise in SUVr at two years, indicating that Aβ deposition has not progressed. Only four of the fifty-nine Aβ-negative individuals at baseline underwent transition to Aβ-positive SUVr levels. The authors came to the conclusion that SUVr might be a trustworthy and repeatable biomarker for tracking alterations in Aβ deposition. Later research has produced supporting evidence for this conclusion [141,142] and comparable outcomes are being attained while imaging tau with PET agents [143,144], pertinent to carefully delivering therapeutic treatments that target tau. Similar to cancer, it is likely that the presence of a biomarker is not the only factor in determining whether or not to take action. Instead, it may be necessary to consider the biomarker’s trajectory over time. As a result, it is feasible that some older persons who are cognitively normal and amyloid “positive” will be watched over time to see if their amyloid values change. If they do, intervention will then start to optimize the therapeutic benefit of the medications.

Although it is still unknown whether monitoring variations in Aβ deposits, tau in NFTs, and inflammatory response can be helpful in evaluating the effectiveness of treatment strategies, doing so would enable PM by allowing doctors to customise patients’ regimens, reduce the likelihood of side effects, and have a smaller financial impact on healthcare.

### 4.4. Implementation of Artificial Intelligence as a Road to Precision Medicine

Wearable sensors and “OMICS” or “EXPOsOMICS”-based measurement and evaluation techniques have the tendency to create a lot of data, requiring new digital methods and assets for processing, integrating, and analyzing the enormous quantities of data [145]. Artificial intelligence (AI) is a young field that enables computer systems to perform tasks independent of human supervision. Initially provided by computer systems that are typically categorised, this implies that each data point has a label or annotation that can be recognised by an algorithm, in order to construct an effective AI algorithm. After the algorithm has been given a sufficient number of collections of datasets and their labels; accuracy of the output is verified. These AI algorithms are incredibly effective at monitoring, evaluating large amounts of data, and spotting patterns [146]. In this perspective, AI is comprised of machine learning (ML), deep learning (DL), and artificial neural networks (ANN). When combined with very effective computer techniques, AI enables us to determine and evaluate disease risk based on an individual’s data [147]. Now, ML/AI systems are in charge of turning such massive amounts of data into medical knowledge. On these platforms, promising outcomes in more accurate illness risk prediction have been demonstrated [148,149,150]. As AI permeates the field of PM, it has the potential to both expand our understanding of the causes and progression of chronic diseases and assist organisations in maximising its benefits.

AI-based systems have already demonstrated improvements in diagnostic performance and accuracy across a variety of specialties, particularly in radiology [151,152,153]. Numerous AI systems have been granted a license by the US Food and Drug Administration (FDA) to advance medical imaging assessment, such as the identification of aberrant lesions that may develop into cancer [153]. A recent study demonstrates how the goal of personalised PM is empowered by AI and the development of technologies as a whole [154].

Data types are integrated, and their associations are examined in a number of ways, and ML is essential for the integration of multiomics data [155]. The UK Biobank project, one of the major prospective cohort studies, has gathered comprehensive phenotypic and genetic data from 500,000 people, encompassing biological assessments, lifestyle variables, urine and blood biomarkers, and brain imaging. This initiative gave researchers the chance to look for genetic links to disease risk, and it produced multiple papers [156]. A PM screening study that presented a platform of profound quantitative multimodal phenotyping that included genomics, advanced imaging, metagenomics, metabolomics, clinical testing, and history of family also offered a thorough, predictive, and individualised assessment of people’s health and chronic disease risk [157].

In a recent study, in addition to the huge data gleaned from deep phenotyping, participants also had access to a behavior coach. The Pioneer 100 Wellness Project (P100) represented a first attempt to collect and examine substantial omics data- sets in order to link molecular circuits in 108 healthy individuals. Over more than a nine-month timeframe, this study did whole genome sequencing, microbiome, proteome, and metabolome analyses. It also recorded clinical data, daily physical activity, and sleep patterns. Each participant had a personal, comprehensive, dynamic data cloud generated by the researchers, who also conducted an integrated study of six various kinds of data. Furthermore, the participants’ wellbeing in terms of diet, inflammation, diabetes, and CVDs was dramatically improved by these data-driven insights combined with behaviour coaching [158]. For 109 people who were at a higher risk for diabetes, a similar study used wearable monitoring and extensive longitudinal omics profiling. They used multi-omics, including measurements of the gut microbiota and the genome, transcriptome, immunome, metabolome, and proteome. They identified 67 clinically actionable outcomes over such a lengthy period, such as the risk of cardiovascular disease [159].

## 5. Limitation, Regulation, and Ethical and Societal Consideration of Precision Medicine

While innovations are being used to create a new approach to decision-making, PM has not been assured to be valuable or effective due to a lack of information, strong clinical evidence, and widely accepted data models for validating the practical work [160].

Considering the availability of different data structures and associated challenges in implementing the current data interchange standards, there have been numerous restrictions and difficulties in medication, laboratory, diagnostic, imaging, pathophysiology, clinical findings and outcomes, and protocols, such as incorporating genetic results in a searchable manner into electronic medical records (EMR). Additionally, testing that is carried out in outside labs frequently cannot be incorporated into other networks. Some of the major issues with data exchange are variations in lab coding systems and identifications or missing genetic information in electronic health record (EHR) datasets [160]. Additionally, selecting the ideal time for clinical data sharing develops into a competitive procedure that requires attention [161].

Data management has not yet been able to keep up with the speed at which a lot of information is produced from numerous sources. Such data require technology and software facilities for analysis and processing, which go beyond the capabilities of many small laboratories and their local infrastructures. Strong computational techniques are required for the processing of molecular and omics information in particular. The maintenance costs and progress are considerable for connected facilities and component resources [162]. To combat the growing expense of storage and computational demands, embracing technologies such as Internet of Things (IoT) and cloud computing can be a solution. Another difficulty is balancing data security, privacy, and related ethical concerns. According to estimates, the healthcare sector is 200 percent more likely than other sectors to face a data breach. Furthermore, ethical concerns prohibit an open data exchange because data produced modularly via the internet is frequently held offline [162]. When discussing ethical dilemmas, for instance, unauthorized access and information misuse received through illegal access present significant obstacles [163].

In most cases, PM entails the investigation of a prognostic biomarker that suggests a favourable therapy relationship. In order to determine whether biological and statistic interactions are connected, pharmacological activity is frequently explored in pre-clinical research and recommended, or early clinical phases may use surrogate endpoints. However, it is often still difficult to show a real (and pertinent) interaction with regard to the clinically important outcome. Interaction on a particular statistical scale may just be caused by the scale’s selection but does not necessarily entail the existence of a biological connection. As per the standard regulatory paradigm, a drug’s usefulness in the population is intended to treat and must be independently confirmed in a typically large Phase III trial without depending on prior research. In this regard, drug approval asks for efficacy and acceptability in the biomarker-defined subset but does not always depend on a complete demonstration of the value of the limitation to a specific population. A significant treatment-by-subpopulation interaction is frequently not powered to be detected in clinical trials with strict clinical endpoints, which is further compounded by the several other causes of variability. If many measurements for each patient are difficult or impossible to make and may be confused by a patient-by-treatment relationship, it is unlikely that the existence of a variance within a patient would be detected. The ideal situation pits those patients who will benefit most from treatment against those who are less likely to do so. However, it can be difficult to distinguish this situation from patients who all have a moderate chance of responding. Therefore, there is a lot of work needed to investigate and validate plausible prognostic biomarkers. In AD, it may be difficult to validate predictive biomarkers using clinically meaningful criteria. On the other hand, there are currently no identified preliminary surrogate endpoints that might be used to assess the biomarker-based candidate identification and are capable of predicting the therapeutic efficacy in clinically meaningful endpoints. Therefore, a challenge for future study is the examination of predictive biomarkers aimed to characterise a receptive population. Similar to other treatment fields, there is an urgent need for medications that are very beneficial in a specific subset of the patient population. However, justifying the selection is difficult and calls for greater data and a thorough knowledge of the root causes of variability. However, the use of biomarker-guided therapies to reduce specific patient subpopulations is well-established in the field of cancer. The use of biomarker-based application during trial proceedings has also been shown in numerous instances in the cancer sector to significantly shorten the period between drug development and clinical application. Consequently, although difficult, there is a proven model for regulatory approval which can be used.

Along with organisational, medical, technological, and scientific problems, the actual use of a PM-based treatment for patients with dementia and those with pre-dementia presents significant ethical, legal, and societal issues. Considering recipients’ assessments of the dangers and benefits of a novel medical treatment will undoubtedly have an impact on how widely it is accepted. First, the application of PM to the study of neurodegenerative disorders would profoundly alter the way we classify theoretical disorders into simple, reductionistic categories, ultimately undermining the notion of what constitutes “healthy” vs. “pathological” [164] into a more dynamic and diversified dimensional idea of diverse genetic and biological of pathophysiologies. Nevertheless, the patient may elect to undergo testing for genetic and biomarkers for initial risk analysis and recognition of a presumably incurable disease including neurodegenerative diseases. In this case, significant ethical choices and even more difficult and complex interactions between patients and doctors and contract procedures are ultimately presented. The employers and insurance firms may access private data and information which could result in “genetic or biological” inequality, which is a second ethical concern presented by PM in the domain of dementia [165]. Another worry is the issue of data rights and informed permission, when storing and utilising patient data in massive databases [166], regarding privacy, security, secrecy, and legal or constitutional rights to one’s own persona. To achieve this, political viewpoints and regulatory measures will direct the way privacy issues are addressed, either toward a supported model of open data or towards selectively restricted access to anonymous data, and a gatekeeper protecting and preventing it from being reidentified [167]. In the realm of dementia disorders and neurodegenerative diseases, the use of PM will place a tremendous emphasis on security, accountability, and transparency practises. The main obstacles to PM include issues with science and technology, security, the advantages of “omics” testing, technological development and assessment techniques, associated ethical concerns, and the collection and use of socially relevant data [168]. Ironically, a significant global collaborative effort is unquestionably essential for the achievement of an individual-centred approach [168] getting multiple stakeholders involved (caregivers, academics, payers, regulators, governments, plan makers, and people in general). A complex governance and multidisciplinary environment where the lines between studies, healthcare, world affairs, and community are blurred may regrettably and inevitably result in conflicts of interest and misinterpretation in the early stages due to the several stakeholders with different aspirations, interests, and various degrees of science literacy. In order for the information gained using tests and techniques to be correctly transmitted and interpreted comprehensibly to the general practitioners and the general community, more advancements in data monitoring and assessment tools are also required. Future applications of PM will need to pay more and more attention to these various ethical concerns, including societal acceptance and cost-effectiveness. To finally allow universal and customised applications, a coordinated approach is required to give extensive public provision for studies, participation in investigations, and eventually adoption of PM in the area of neurodegenerative diseases. Indeed, PM in neuroscience, neurobiology, and psychology is still a courageous vision that lies far beyond the frontier of existing views. It is needed to combine genetic and biological records with phenotypic, cultural, societal, and private priorities and lifestyle to deliver a more individually tailored treatment and prevention of biochemical pathways that eventually lead to cognitive dysfunction while vitally considering moral, sociocultural, governmental, and public perspectives.

## 6. Future Prospective to the Usage of Precision Medicine

To expeditiously spread the use of the PM paradigm [42] to a wider range of complicated illnesses [169], the implementation of Precision Medicine Initiatives (PMIs) is backed by numerous governments worldwide. These are significant initiatives that seek to produce the vast body of scientific knowledge required to enable revolutionary advancements in early detection, prophylaxis, and treatment as well as to effectively implement the concept of PM into routine clinical practice [170].

U.S. President Barack Obama disclosed the Precision Medicine Program Cohort Program on 20 January 2015, a research initiative designed to hasten the transition to a new generation of PM (PMI-CP) that is available at Precision Medicine Initiative | The White House (https://www.archives.gov/, accessed on 1 July 2022) that is anticipated to draw in a research cohort of more than a million Americans. This demographic will be asked to consent to detailed biological specimen and behavioural characterization that is all related to EHRs. It will be possible to conduct observational studies of medications and technologies, as well as potentially assist more rigorous interventional research addressing particular issues, thanks to the systematic collecting of deep, vast, and complicated data [170]. This strategy is expressly intended to target brain diseases such as AD, although initially concentrating on significant disease areas, including cancer. Cross-sectoral partnerships and multidisciplinary research are meant to clarify how neurodegenerative illnesses arise and more effectively integrate new knowledge into preventive and treatment methods. Some PMIs include provisions for research in regulatory disciplines and seek to include administrations and larger populations in technology advancement. The idea of open and accountable innovation strikes a balance between the demands for investment, regulation, and expanding access to potential cures and diagnostics for neurodegenerative disorders. Utilizing both proven and cutting-edge methods to collect and handle enormous amounts of complicated data will be essential for the successful establishment of the PMI-CP [169]. This will be made possible by developments in information technology, which have significantly decreased the cost of data preservation and similarly increased analytic capabilities, enabling the creation and analysis of enormous clinical databases in the field of biomedicine.

Along with technology advancements, patients are now negotiating with clinicians and researchers. They have become more involved in and active in the healthcare system; they are more associated and coordinated through networks; and finally, they have become much more “impatient” as they keenly seek out improved treatments for either themselves or the individuals they care about. Researchers can interact with caregivers, patients, and advocacy groups to assemble patient-linked omics and SB records in order to expand clinical trials, to expedite proactive communication with government regulators, to help in the explanation of therapeutic value, and to ensure that PMIs can comply with the requirements of patients. These elements point to a crucial transformation in culture, ethics, and thought that is necessary for precision medicine to succeed. However, the availability of medical proof of beneficial clinical outcomes and comparable costs must be taken into account [171]. With the application of appropriate assets and a continuous commitment of efforts, time, expertise, and skill from the research and medical communities, it is without a doubt possible to ultimately realize the full potential of PM.

The future of PM in AD is promising, as research continues to identify new biomarkers and targeted therapies. The use of PM has the potential to improve the accuracy of diagnoses, leading to earlier intervention and potentially better outcomes. One promising area of research is the use of genetic biomarkers to classify subtypes of AD, which would allow for more tailored treatment approaches. Additionally, new imaging techniques, such as positron emission tomography (PET) scans, are being used to identify biomarkers that indicate the presence of amyloid plaques and tau tangles in the brain. PM also has the potential to improve the development of new treatments for AD. By identifying specific subtypes of the disease, researchers can focus on developing therapies that target the underlying causes of the specific subtype, potentially leading to more effective treatments with fewer side effects. Another important prospective is the use of machine learning techniques in PM. This can help in the early prediction of AD and make for a more accurate diagnosis [172,173,174,175]. In summary, precision medicine has the potential to revolutionize the way AD is diagnosed and treated, leading to more effective therapies and improved outcomes for patients.

## 7. Conclusions

There are rising paradigm changes in how we think about medical knowledge and research. Furthermore, the discipline of medicine is at a turning point. The convergence of advances in techniques and theories, including SB, genomic sequencing, explorative high throughput assessments, the advent of biological markers, records and computational methods, interconnected disease modelling, EHRs, intelligent systems, and P4 medicine, has undoubtedly made PM one, if not the main, innovative goal. The PMI-CP, which U.S. President Obama announced in 2015, is significant in this context. It is estimated that about a million US citizens will take part in this programme by contributing their genetic information, biological material, and behavioural data, which will all be carefully examined and afterwards integrated to EHRs. The highly detailed data that SB will produce will be crucial for analysing the underlying molecular mechanisms of numerous common complicated diseases, which will pave the way for the creation of personalised, biomarker-guided treatments that are both effective and safe. These enormous strides toward reintegration into medicine and acceptance of PM as the main focus of coordinated efforts have not yet been taken by the fields of psychiatry, neuroscience, and other related fields. As we can learn from the developments in cancer, the PM paradigm has to be adopted and applied more quickly for improved health results that will have a significant effect on global economic results. As a result, PM in the domain of AD and neurodegenerative disorders should focus on genetic risks and the earliest preclinical asymptomatic stages of the disease, when the condition is treatable. These strategies should be designed to delay, halt, and feasibly prevent the development to clinical manifestations. In order to globalise, standardise, and revolutionise the current approach to scientific and clinical neurological research, it is imperative that citizens (who are active participants in studies and are no longer considered “patients”) and policy makers work more closely with caretakers, basic researchers, and medical scientists. This inevitably necessitates an extreme conceptual and ideological shift away from traditional notions, which are based on the intervention of late-stage illnesses led by diverse clinical characteristics treated by speculated “one-size fits all-magic bullet therapies”, to the patient-centered PM-based strategy, centred on initial risk screening and detection with tailored and biomarker-guided treatments to accomplish safe and effective prevention and therapy. Through international, multidisciplinary collaborative efforts, the PMI’s aim and goal is to support a radical shift and revolution of research towards PM. Table 2 enlists some international projects associated with PM and its objectives [176].

## Figures and Tables

**Table 1:** AD Biomarkers. AD: Alzheimer’s disease; CSF: Cerebrospinal fluid; Aβ: Amyloid beta; APP: Amyloid precursor protein; NFL: Neurofilament light chain protein; NSE: neuron-specific enolase; VLP-1: Visinin-like protein 1; HFABP: Heart fatty acid binding protein; MCP-1: Monocyte chemoattractant protein-1; GFAP: Glial fibrillary acidic protein; sTREM2: Triggering receptor expressed on myeloid cells 2. Data obtained from [127].

S.No.	Biological Marker	Description	Depiction	Cerebrospinal Fluid	Diagnostic Efficiency
1.	Aβ42/Aβ40	Aβ involves the processing of the amyloid precursor protein (APP) by enzymes called β-secretase and γ-secretase. These enzymes cleave APP to form Aβ peptides of varying lengths, with the most common being Aβ40 and Aβ42. The Aβ peptides can then aggregate to form amyloid plaques.	APP metabolism marker	A lower level is present in AD patients.	Indicated for diagnosing CSF.
2.	Aβ38	Aβ38 is a specific form of the Aβ peptide. Aβ peptides are generated through the cleavage of the APP by enzymes called beta-secretase and gamma-secretase. Aβ38 is a less common form of the Aβ peptide compared to the more prevalent Aβ40 and Aβ42.	APP metabolism marker.	No variations between groups.	Not really helpful on its own. May be useful in separating AD from dementias that are closely similar to AD.
3.	sAPPα	sAPPα (soluble amyloid precursor protein alpha) is a fragment of the APP that is generated by the action of the α-secretase enzyme. This enzyme cleaves APP at a different site than the β-secretase and γ-secretase enzymes, which results in the production of different set of peptides.	APP cleavage product.	No variations between groups.	Not really helpful on its own.
4.	sAPPβ	sAPPβ (soluble amyloid precursor protein beta) is a fragment of the APP that is generated by the action of the beta-secretase enzyme. This enzyme cleaves APP at a specific site, releasing sAPPβ and the C-terminal fragment (CTF) of APP.	APP cleavage product.	No variations between groups.	Not really helpful on its own.
5.	t-Tau and p-Tau	Tau protein is a microtubule-associated protein that is found in neurons and is important for the stability and function of microtubules.	Markers connected to memory difficulties.	A higher level is present in AD patients.	P-tau is a hallmark of AD. Indicated for diagnosing CSF.
6.	NFL	NFL protein is a type of intermediate filament protein that is found in the neurons of the nervous system. It is an important component of the cytoskeleton, which provides structural support for the cell. NFL protein is part of a group of neurofilament proteins that also includes neurofilament medium chain (NFM) and neurofilament heavy chain (NFH) proteins.	Neurodegeneration-related biomarker.	A higher level is present in AD patients.	Indicated for diagnosing CSF.
7.	NSE	NSE is a protein that is found in high concentrations in neurons and neuroendocrine cells. It is an enzyme that plays a role in the metabolism of glucose and is considered to be a marker of neuronal damage.	Neurodegeneration-related biomarker.	A higher level is present in AD patients.	Possibly helpful for diagnosing CSF.
8.	MCP-1	MCP-1 is a small protein that is known to be involved in the recruitment of immune cells, specifically monocytes, to sites of inflammation and injury. MCP-1 may contribute to the accumulation of amyloid beta, which is a hallmark of AD.	Glial activation marker.	A higher level is present in AD patients.	Not really helpful on its own.
9.	VLP-1	VLP-1 1 is a protein that is found in the retina and is a member of the visinin-like protein family. VLP-1 is expressed in the inner segments of rod and cone photoreceptor cells, where it is involved in the regulation of intracellular calcium levels. VLP-1 is also found in the brain, where it may have a role in synaptic plasticity and learning.	Neurodegeneration-related biomarker.	A higher level is present in AD patients.	Possibly helpful for diagnosing CSF.
10.	HFABP	HFABP is a small, cytosolic protein that is found in high concentrations in the heart and other tissues. It binds long-chain fatty acids and is involved in the intracellular transport and metabolism of fatty acids.	Neurodegeneration-related biomarker.	A higher level is present in AD patients.	Possibly helpful for diagnosing CSF.
11.	GFAP	The accumulation of Aβ and tau protein, which are the hallmarks of AD, are known to cause astrocyte activation and increase the expression of GFAP. This suggests that astrocyte activation may play a role in the development of AD.	Glial activation marker.	No variations between groups.	Not really helpful on its own.
12.	Neurogranin	Neurogranin is a protein that is primarily found in the brain and is thought to play a role in synaptic plasticity and memory formation. It’s a postsynaptic protein and is found to be associated with N-methyl-D-aspartate (NMDA) receptors, which are important for synaptic plasticity and memory formation.	Synapse degeneration marker.	A higher level is present in AD patients.	Particular to AD. High potential but little published studies.
13.	α-Synuclein	α-synuclein is a protein primarily found in the brain and it’s known to be involved in the regulation of neurotransmitters release and synaptic function.	Protein at presynapse.	A higher level is present in AD patients.	Not really helpful on its own. Most studies are carried out with likely AD individuals.
14.	sTREM2	The accumulation of Aβ, which is a hallmark of AD, is known to activate microglia, and it is thought that the increased sTREM2 levels may be a result of this activation.	Neurodegeneration-related biomarker.	A higher level is present in AD patients.	Possibly helpful for diagnosing CSF. Little published studies

**Table 2:** Projects being implemented worldwide to use precision medicine.

Name of the Project	Objective	Country
Australian Genomics Health Alliance	Create a national framework for integrating guidance on reporting results from clinical testing and genomics research into clinical study and experimentation.	Australia
Belgian Medical Genomics Initiative	Anticipate health outcomes using genomic data and carry out a pilot project in Belgium to integrate genomic data into clinical care.	Belgium
Genome Canada	Conduct extensive studies examining the use of genomics in the field of precision medicine. A decision-based, evidence-based strategy to healthcare and general health might be described as precision health.	Canada
Estonian Program for Personal Medicine	Sequence 5000 individuals, create an Estonian genotyping array, test it on 50,000 Estonian Biobank participants, make it available to all adults aged 35 to 65 (about 500,000 individuals), and connect it to the EMR.	Estonia
Plan France Medecine Genomique 2025	Utilize the combination of patient care, education, and research to provide everyone with access to genetic medicine.	France
Bench-to-Bedside Project	100,000 Israeli genomes from chosen patients will be sequenced.	Israel
Implementation of Genomic Medicine Project	Utilize genomics for the best possible diagnosis, care, and prevention.	Japan
Genome Technology to Business Translation Program	Subsequent developments treatment and diagnosis methods for personalised and preventive medicine using genomes.	Korea
Centre for Systems Biomedicine	Prioritize early Parkinson disease diagnosis and classification.	Luxembourg
Personalized OMIC Lattice for Advanced Research and Improving Stratification	Develop a 90-gene panel for gastrointestinal malignancies after implementing a TGFBI gene testing pilot for diagnosis of diseases and family risk evaluation in stromal corneal dystrophies.	Singapore
Pharmacogenomics Network	Use pharmacogenomics cards in conjunction with a national pharmacovigilance programme to determine the risks.	Thailand
Genomics England	To better comprehend cancer, uncommon disorders, and infectious diseases, 100,000 whole genomes will be sequenced and linked to National Health Service information.	United Kingdom
All of Us Research Program	To advance scientific research and clinical treatment, enrol one million participants who are representative of the community and share data from their EMRs, digital health technologies, and genomics.	United States

## Data Availability

Not applicable.
